# Supplementary material for: Impact of school closures and reopening on COVID-19 caseload in 6 cities of Pakistan: An Interrupted Time Series Analysis
Source: PLOS Glob Public Health. 2022 Sep 19;2(9):e0000648. doi: 10.1371/journal.pgph.0000648 (PMC10022346; doi:10.1371/journal.pgph.0000648)
Supplement: S1 Table — (PDF) [file pgph.0000648.s001.pdf]

S1 Table. Non-school closures non-pharmaceutical interventions (NPIs) during study period (November 6, 2020 to March 22, 2021)

City	Date of marriage-hall restrictions	Date of smart/micro smart lockdowns at hotspot areas
Lahore	November 20, 2020	November 10, 2020
Karachi	November 20, 2020	November 21, 2020
Islamabad	November 20, 2020	November 9, 2020
Quetta	November 20, 2020	November 19, 2020
Peshawar	November 20, 2020	November 7, 2020
Muzaffarabad	November 20, 2020	N/A
