# Supplementary material for: Impact of school closures and reopening on COVID-19 caseload in 6 cities of Pakistan: An Interrupted Time Series Analysis
Source: PLOS Glob Public Health. 2022 Sep 19;2(9):e0000648. doi: 10.1371/journal.pgph.0000648 (PMC10022346; doi:10.1371/journal.pgph.0000648)
Supplement: S1 Text — (DOCX) [file pgph.0000648.s002.docx]

**S1 Text: Methodology steps**

1. We took 60-day periods for our Single Group Interrupted Time Series Analysis (ITSA), for 10- and 20-days lagged school closure and schools reopening periods.
2. We estimated our ITS estimators and kept intervention dates 10-days and 20-days after original dates of school closures (November 26, 2020) and schools reopening (February 1, 2021) for each city. We took the following steps in our analysis:
   1. Labeled and converted data to categoric and specified the data as time series
   2. Described of summary statistics
   3. We ran our ITSA models using itsa command in Stata, provided by Linden Consulting.
   4. Newey-West standard errors were used, which can correct for possible heteroskedasticity and auto-correlation. In OLS regressions with Newey-West standard errors, correct lag structures were specified for unbiased estimates through Cumby-Huizinga test for autocorrelation.
   5. Afterwards, linktest was applied for model specification. An insignificant _hatsq value showed our models were correctly specified.
