# Supplementary material for: Impact of school closures and reopening on COVID-19 caseload in 6 cities of Pakistan: An Interrupted Time Series Analysis
Source: PLOS Glob Public Health. 2022 Sep 19;2(9):e0000648. doi: 10.1371/journal.pgph.0000648 (PMC10022346; doi:10.1371/journal.pgph.0000648)
Supplement: Attachment — Submitted filename: Response to Reviewers.docx [file pgph.0000648.s005.docx]

**Reviewer # 1:**

1. “Please describe your reasons for choosing only provincial capitals for data analyses?”

- **Response:** We selected only provincial capitals since these were the largest cities in provinces with the highest COVID-19 caseloads during the analysis period. We have now included the reasoning in first paragraph of methodology of the paper as well (lines 74-77).

2. “why only 60 days of window pre and post for data analyses?”

- **Response:** A one-month window for each pre- and post-intervention period was chosen since the trends were not changing in this period other than because of the interventions of school closures and reopening. Box and Jenkins recommend at least 50 time points for time series analysis and power increases as sample size increases. However, we did not take a longer time period than this as schools had started to reopen after one-month post-intervention in our 20-days delay model. A clarification has been added in the methodology now (lines 114-118).

3. “please provide which schools were included and why??”

- **Response:** We did not disaggregate the data on school level as it was not possible. Instead, our analyses is at city-wide level which included all schools in those cities. The terms “School closures” and “School reopening” refer to all schools – public or private, day or boarding – in the named cities. We have clarified this in the second paragraph of methodology section (lines 89-91).

**Reviewer # 2:**

Specific Comments: Much of your arguments for writing this paper hinges on the second paragraph in the methods section. I don’t understand the second sentence in that paragraph. Is it saying that all cities also had several restrictions at the same time as was the case with school closures? It seems to me that everyone applied all the programs at the same time.

- **Response:** Yes, that is correct, all other interventions were being applied equally across all cities and at the same time, using the same implementation mechanism and measurement metrics. In the last line of paragraph above this, we say that ITSA is used where we need to consider the effect of only one intervention. We justify the rationale behind using ITSA for school closures in the first and second sentences of second paragraph by saying that all other policies/NPIs were notified and enacted in groups (e.g., marriage-hall restrictions and ban on large gatherings were notified at the same time). Therefore, we cannot isolate the effect of any of these NPIs which were implemented in groups. But school closures were notified in isolation so we can exploit that as a natural experiment to try to estimate the association with COVID-19 cases.

Are you saying that school closures fluctuated during this time in all six cities where as other measures did not? I would like to see a greater defense of the line that NPI is the only policy intervention that could be isolated in your chose time period of observation to be consistent across six counties.

- **Response:** We have added explanation for this in methodology second paragraph (lines 84-91) and also created a table S1 which shows what other policies were put in place during this time.

So, from Table 1, should we understand that all other programs do not exist during these times?

- **Response:** Table 1 only shows intervention dates for school closures and reopening that we take in our analyses. We have clarified the table title now.

Auger et al. paper incorporates a lag period to allow for potential policy-associated change to occur. Your reasoning for delays is different, 1st line 4th para in Methods section.

- **Response:** Auger et al. calculate lag periods themselves. We rely on prior research for choosing the number of delays for school closures’ potential effects to occur.

You have only six cities, can you state what other policies might be in place during these times? You could introduce a table stating what was in place during the times listed in Table 1 for each city.

- **Response:** We have added S1 table in Supplementary material for other NPIs that were implemented during the period of our study.

“Can you state how reliable is your data. Please justify the data set and also state its limitations.”

- **Response:** The data for this study was directly sourced from the National Situation Reports (Sitreps) published by the National Emergency Operations Centre (NEOC) in Islamabad, Pakistan. It is the Ministry of Health’s official COVID-19 data that was being analyzed and used during Pakistan’s pandemic response nationally and for international reporting, and hence it is the most reliable one. This data has limitations however, as it is anonymized and aggregated by city, with no disaggregation by age, gender, ethnicity, or any other potentially identifying characteristic. We use this data for an inferential analysis of the change in daily COVID-19 incidence in the overall populations of the 6 cities, regardless of demographic characteristics. We have included this in the methodology (paragraph 3) and limitations sections.

“Your equation specification reads like a panel. You may think of clarifying it. You run six estimations for each city, not a panel.”

- **Response:** As we run our model for each city separately, we do not take it as a panel. We have now clarified this in Model Specification heading (lines 142-144).

“It would be nice to have graphical representation of all six cities and also the country using the raw data.”

- **Response:** We have added figures in results section now (lines 204-211).

“You might want to estimate your results in terms of percentages in the discussion section.”

- **Response:** Auger et al. use a slightly different approach for reporting their results in the paper. They report “relative change in the outcome from week to week” of cumulative COVID-19 cases and therefore their results are in percentages. We use Linden’s interpretations cited in our study, i.e., in terms of rates of change of daily COVID-19 cases in the post-intervention periods. In the discussion section, we report actual COVID-19 cases instead of COVID-19 cases per 100,000 population.

“I don’t follow how you obtained the last line in the 1st para of the Discussion section.”

- **Response:** We have removed that line because it used imputed numbers based on the results of this paper, which in itself is another analysis that we do not include in the paper.

“I am puzzled by the reference to 34, the Doug North et al. book. Do you mean social networks contacting each other? It is likely that personal communication between relatives would be higher in Pakistan and in the cities they would be dispersed? Comparison should be with other South Asia and Vietnam as Europe and North America have much greater resources.”

- **Response:** In second paragraph of discussion section, we compare Pakistan’s caseload with other countries, primarily developed. We provide possible reasons of Pakistan’s lower cases, one of them being sparser social networks in Pakistan as compared to developed countries. Doug et al. argue that rich countries with more economic development have more and deeper social networks than poor countries, and since Pakistan has evidently a lack of economic development, it implies that it has lesser and sparser social networks than Europe or North America.

“I am not sure school closure impact is conclusive even in the works by Auger et al. Identification strategy amid so many other restriction is suggestive at best, especially since alternate policies were likely to be many during these times.”

- **Response:** Thanks for pointing this out as we acknowledge that in our conclusion that school closures are maybe associated with lower transmission of cases, without implying strict causation. In discussion section paragraph 4, we only use the word “association” of school closures with reduced COVID-19 transmission as the impact is, as you pointed out, suggestive to an extent.
